# Translocation of Tebuconazole between Bee Matrices and Its Potential Threat on Honey Bee (*Apis mellifera* Linnaeus) Queens

**DOI:** 10.3390/insects13010045

**Published:** 2021-12-31

**Authors:** Risto Raimets, Sigmar Naudi, Marika Mänd, Vadims Bartkevičs, Guy Smagghe, Reet Karise

**Affiliations:** 1 Institute of Agricultural and Environmental Sciences, Estonian University of Life Sciences, F. R. Kreutzwaldi 1, 51006 Tartu, Estonia; sigmar.naudi@emu.ee (S.N.); marika.mand@emu.ee (M.M.); reet.karise@emu.ee (R.K.); 2 Institute of Food Safety, Animal Health and Environment “BIOR”, Lejupes Street 3, LV-1076 Riga, Latvia; vadims.bartkevics@bior.lv; 3 Department of Plants and Crops, Ghent University, B-9000 Ghent, Belgium; Guy.Smagghe@ugent.be

**Keywords:** translocation of pesticides, wax, hazard quotient, tebuconazole, honey bee queen

## Abstract

**Simple Summary:**

Numerous pesticide residues have been found in bee products. It is unclear whether and to what degree pesticides migrate between different bee matrices. Even though the use of many common insecticides is strictly regulated, fungicide residues are still ubiquitous in bee matrices and data regarding this problem are still insufficient. The aim of this work was to determine the migration of fungicide tebuconazole between bee matrices and to assess its potential risk to honey bee queens. We found that tebuconazole mixed into wax has the potential to migrate into royal jelly (RJ), but no residues were found in honey bee queen larvae and newly emerged queens. The residues of tebuconazole found in queen cell cups and RJ decreased over time and probably posed no direct lethal threat to queens. Nevertheless, sub-lethal effects of tebuconazole on honey bee queens might occur even at low concentrations.

**Abstract:**

Various pesticide residues can be found in different bee colony components. The queen larvae of honey bee (*Apis mellifera* L.) receive non-contaminated food from nurse bees. However, there is little knowledge about how pesticide residues affect developing bees. Additionally, little is known about the migration of lipophilic pesticides between bee matrices. While wax, royal jelly (RJ), and bee larvae are chemically distinct, they all contain lipids and we expected the lipophilic fungicide tebuconazole to be absorbed by different contacting materials. Our aim was to analyze the translocation of tebuconazole residues from queen cell wax to RJ, queen larvae, and newly emerged queens and to evaluate its potential risk to queens. We demonstrated the potential for the migration of tebuconazole from wax to RJ, with a strong dilution effect from the original contamination source. No residues were detected in queen bee larvae and newly emerged queens, indicating that the migration of tebuconazole probably did not directly endanger the queen bee, but there was some risk that tebuconazole might still affect the homeostasis of developing bees.

## 1. Introduction

Pesticides are known to be amplify all stress elements related to honey bee colony losses [1]. Various pesticides accumulate in different bee products [2,3] and often beeswax is the most contaminated bee product [3,4,5]. Most of the conducted studies have been focused on insecticide effects on bees, while fungicides have gained little attention. Nevertheless, it has been shown that at least azole type fungicides can potentiate the toxicity of insecticides [6,7,8].

Azole fungicides are among the highest volume fungicides used against fungal diseases of agricultural crops [9]. Among other azole fungicides, tebuconazole is very commonly used and its residues have been found in bee matrices [2,10,11]. Tebuconazole has synergistic negative effects on honey bees together with insecticides [10]. Additionally, it can separately affect microbial communities in different environments, including those in insect guts [11,12].

The large majority of bee toxicology studies have been focused on the impact of pesticide residues on worker bees [13,14,15], while queens have received little attention. The main task of a mated queen is to sustain colony development and survival via laying eggs [16]. During their lifetime, queens are fed pure royal jelly (RJ) secreted by nurse bees [17] that feed on nectar, pollen, and beebread, which can be contaminated by various pesticides [3,18,19,20]. Contaminated pollen and nectar can lead to contamination of beeswax, which absorbs lipophilic compounds well [3,5,21,22]. This may pose long-term risk to the viability of a beehive. Although the queen bee is protected from xenobiotic compounds due to feeding solely on RJ, it spends most of its life on or in wax combs. It has been shown that honey bee larvae can suffer morphological changes when grown on contaminated wax [23]. The same changes may occur in queen larvae, because the ambient conditions are the same during queen development.

Long-term contact with pesticide residues exposes bees to a certain risk. One way to quantify the potential risk is to calculate a hazard quotient (HQ) [21]. HQ is calculated by dividing the concentration of contaminant in wax with the LD_50_ value (the concentration that would kill 50% of the test group individuals) of the substance. HQ simply evaluates the risk of individual contaminants to bees. However, depending on their specific tasks in colony, the individual honey bees are in contact with different materials [3]. In the case of multiple contaminants, the calculation of HQ still helps to understand the contribution of individual contaminants to the overall risk.

The contaminating molecules are able to migrate between materials inside the hive [22]. The migration ability depends on the chemical nature of the contaminant, on the chemical and physical characteristics of the material.

The information of pesticide migration among bee products is still scarce. For instance, Böhme et al. showed that RJ is not contaminated [24], despite the contaminated pollen they were fed with. Similar results were shown by Johnson and Percel, where nurse bees that were fed contaminated pollen secreted pure RJ [25]. However, contamination of RJ can occur via exposure to wax comb cells.

The purpose of this study was to test whether tebuconazole residues mixed into honey bee wax migrate from one bee product to another (RJ; queen bee larvae; newly emerged queens). The aim thereafter was to evaluate the potential hazard to queens.

## 2. Materials and Methods

### 2.1. Honey Bees Used

The experiments were conducted in summer 2020 at a single apiary (OÜ R-honey) located in the Eastern region of Estonia. Queens (*Apis mellifera ligustica*) were bred in the experiment from one-day old larvae originating from a single queen. Queenright normal-sized honey bee colony (50 Langstroth frames) was used as a cell builder. An egg laying queen was allocated to the first Langstroth box on the hive bottom and separated with queen excluder. In addition, an extra flight entrance was installed above the queen excluder to encourage building up the queen cells by nurse bees. Honey supers were allocated on the extra flight entrance and the grafted queen cells with open brood frames were all inserted into the top box.

### 2.2. Exposure to Tebuconazole

Wax obtained from a local organic beekeeping operation was used for making queen cell cups. The active ingredient tebuconazole (purity 99.3%) was purchased from Sigma Aldrich. Tebuconazole was dissolved in acetone and incorporated into molten wax. The tebuconazole concentration mixed into wax was 412 µg kg^−1^. Field-realistic pesticide concentration was selected based on the analysis of Estonian bee products by Raimets et al. [26]. In addition, tebuconazole has been found in honey bee wax in other studies [2,3,6].

Immediately after the mixing of tebuconazole into molten wax, queen cell cups were prepared using special wooden dowel to shape the cup. Separate dowels were used for the control and test group cups. It is a common procedure in beekeeping to make queen cell cups from molten wax [26]. One-day-old honey bee worker larvae were grafted into the newly made cups. A frame with the grafted cups (control cups (*n* = 20)) and tebuconazole spiked cups (*n* = 20) was inserted into queenright cell builder colony. After 24 h, the queen cell acceptance by nurse bees was controlled visually.

The closest conventionally managed agricultural crops were located 5.4 km away. The experiment was conducted in July, which is the main honey flow period in Estonia. At that time, there is abundance of plants blooming in the wild. Considering the distance between the apiary and conventional farming fields, it is very unlikely that the bees visited conventional farming fields and were exposed to tebuconazole from outside environment.

### 2.3. Pesticide Residues in Bee Matrices

In order to investigate whether tebuconazole can migrate from one bee product to another, we collected queen cell cups, RJ, accepted queen larvae, and newly emerged queens. RJ and larvae were collected from cups 3 days after the acceptance. RJ was collected using micropipette and larvae were taken from the cells using a special spatula. Adult queens and built-up queen cell cups were taken for pesticide residue analysis on the day of their emergence. All the samples were put into freezer (−20 °C) immediately after the collection. While large volume experiments with honey bee queens are difficult, we could not split the individually treated cells into several groups to obtain the required minimal sample mass (2 g) for pesticide residue analyses. Therefore, the samples were pooled according to the types of bee matrices. The pooled samples were sent to a laboratory (Institute of Food Safety, Animal Health and Environment “BIOR”, Riga, Latvia) for pesticide analysis.

### 2.4. Pesticide Residue Analyses from Bee Matrices

Tebuconazole residues from honey bee matrices were analyzed at the Institute of Food Safety, Animal Health and Environment “BIOR”. The UHPLC-MS/MS assay was performed using an Ultimate 3000 high performance liquid chromatograph (Thermo Scientific, MA, USA) coupled to TSQ Quantiva tandem mass spectrometer (Thermo Scientific, MA, USA).

### 2.5. Chemicals and Materials Used in Pesticide Residue Analyses

Pesticide reference standards were supplied by Dr. Ehrenstorfer (Germany). Methanol and HPLC grade acetonitrile were acquired from Merck Millipore (Darmstadt, Germany). Ammonium formate (99%), acetic acid (glacial, >99.85%), and ACS grade formic acid (>96.0%) were obtained from Sigma-Aldrich (St. Louis, MO, USA). Ultrapure deionized water was prepared by using a Millipore Milli-Q™ system (Billerica, MA, USA). The following substances were purchased from Phenomenex (Torrance, CA, USA): buffer salt mixture (1 g trisodium citrate dihydrate, 1 g sodium chloride, 0.5 g disodium hydrogen citrate sesquihydrate, and 4 g of anhydrous magnesium sulphate) and a mixture of dSPE (900 mg anhydrous magnesium sulphate, 150 mg PSA, and 150 mg C18E). Stock solutions (approximately 1000 mg L^−1^) were made by weighing 10 mg of standard in a 10 mL graduated flask and then dissolving in acetonitrile. While preparing the standard solutions of final concentration, the purity of standard was taken into account. In order to prepare a working standard solution with a concentration of 0.01 mg L^−1^, the appropriate volume of stock solution was diluted with acetonitrile. The prepared solutions were stored at 20 °C.

#### 2.5.1. Sample Preparation

The sample (2.0 ± 0.1 g) was weighed into a 50 mL centrifuge tube. The standard solutions were added at the appropriate spiking level in order to prepare calibration and quality control samples. Acetonitrile (10 mL) and deionized water (10 mL) were both added and the tubes were shaken vigorously by hand for 1 min. Then, a mixture of trisodium citrate dihydrate (1 g), sodium chloride (1 g), disodium hydrogen citrate sesquihydrate (0.5 g), and anhydrous magnesium sulphate (4 g) was added, the tubes were closed, shaken for 10 min, and centrifuged for 10 min at 3500 rpm.

The supernatant was transferred into a 15 mL PP centrifuge tube and frozen out at −70 °C for 30 min using a Heto Ultra freeze (Thermo Fisher Scientific, MA, USA), followed by centrifugation of the resulting organic sample fraction for 10 min at 3500 rpm. For further clean-up procedure, 6 mL of the extract was transferred into 15 mL PP tubes, each containing anhydrous magnesium sulphate (900 mg), PSA (150 mg), and C_18_ sorbent (150 mg). The tubes were shaken vigorously by hand for 30 s and then centrifuged for 10 min at 3500 rpm. A 250 μL aliquot of the purified extract was mixed with 500 μL of the mobile phase A, consisting of 5 mM ammonium formate and 0.1% formic in water and filtered through a 0.22 µm PVDF membrane centrifuge filter. An aliquot of the extract was transferred to an autosampler vial for UHPLC-MS/MS analysis.

#### 2.5.2. UHPLC-MS/MS Analysis

For pesticide analyses in samples, an UltiMate 3000 high performance liquid chromatograph coupled to a TSQ Quantiva tandem mass spectrometer equipped with an electrospray ionization source was applied. The parameters of the ion source were the following: vaporizer temperature was adjusted at 450 °C and ion transfer capillary at 320 °C, ion spray voltage 3.5 kV (positive mode), sheath gas 45 arbitrary units (arb), auxiliary gas 25 arb, and sweep gas 4 arb. The analysis was performed by multiple reaction monitoring (MRM) in the positive ionization mode. Table 1 lists the analyte-dependent parameters: MRM transitions and collision energies (CE).

Chromatographic separation was performed on a Kinetex C18 analytical column (50 × 3.0 mm, 1.7 mm) from Phenomenex. The mobile phase A consisting of 5 mM ammonium formate and 0.1% formic in water and acetonitrile (mobile phase B) were delivered at the flow rate of 0.4 mL min^−1^. A gradient program was used: 20% of mobile phase B was used from 0 to 1.0 min, 20% (B) to 90% (B) from 1.0 to 10.0 min, maintained at 90% (B) for 1 min, then decreased back to 20% (B) at 11.0 min and finally the column was re-equilibrated with 20% (B) from 11.0 to 15.0 min. A 10 µL aliquot of the extract was injected. The column and autosampler were maintained at 30 °C and 10 °C, respectively.

### 2.6. Statistical Analysis

For calculating the hazard quotient (HQ), the wax toxicity calculation tool (Bee Tox Wax) was used [21]. The aim of HQ was to evaluate the potential exposure to a pesticide and to set a level at which no adverse effect is expected. When using the “Bee Tox Wax” tool, the median lethal dose (LD_50_) is a quantitative indicator of the pesticide toxicity. In this case, HQ values under 250 were shown to be slightly toxic to bees and the wax can be re-used.

## 3. Results

### 3.1. The Residues Detected

UHPLC-MS/MS assay showed that tebuconazole was present in certain bee matrices (Table 2). Tebuconazole residues were found in the entire queen cell wax (0.19 mg kg^−1^), indicating that this substance was incorporated into wax during the experiment. The residues were also found in RJ samples but already at lower concentrations. The tebuconazole concentration found in RJ was 0.08 mg kg^−1^ (0.08 µg per bee), which was 2.4 times lower than found in wax and probably should not pose a risk to the survival of honey bee queens. Despite the fact that tebuconazole was present in queen cell cups and RJ, its residues were not found in honey bee queen larvae and newly emerged queens. It could be assumed that queen larvae and adult queens metabolised tebuconazole and thus no residues were found. Nevertheless, the results show the potential for tebuconazole migration from wax to RJ.

### 3.2. Hazard Quotient

According to the findings of tebuconazole residues in wax, the Hazard Quotient (HQ) was used to determine its potential toxicity level to honey bee queens. The HQ value of initial concentration mixed into wax was 1.

The tebuconazole concentration found in queen cells was 0.19 mg kg^−1^ (0.19 µg per bee), which means that it should not cause direct mortality of bees, but this sub-lethal concentration may have significant negative effect on queen homeostasis. In the case of tebuconazole, 200 µg per bee has been shown to be the acute lethal contact dose after 48 h. The HQ value in our case was 1, which means that the wax was slightly contaminated and it can be used in beekeeping and recycling. According to the Bee Tox Wax tool, any HQ value under 250 is considered to be low-polluted and the wax could be used and recycled in beekeeping operations [21]. At the same time, it should be taken into account that the risk for bees will increase with increasing number and levels of different contaminants.

## 4. Discussion

This study has demonstrated the migration of tebuconazole from wax to RJ. The only source of tebuconazole was the addition of this xenobiotic compound to molten wax. However, further migration from RJ to queen larvae or newly emerged queens did not occur. Despite the high initial concentration, the content of tebuconazole detected from the entire queen cell wax was lower.

Lipophilic pesticides like tebuconazole can accumulate in wax [3,27] and, due to continuous agricultural application, the amount of residues in bee matrices can be expected to increase with time. Little is known about the effects of certain pesticides on queen bee performance. In addition, various studies show that different pesticides can cause synergistic effects in bees. Raimets et al. showed that azole fungicide imazalil had a synergistic effect with three insecticides on the mortality of bumble bee (*Bombus terrestris*) [6]. Besides, Vandame and Belzunces showed that insecticide deltamethrin had a synergistic effect with azole fungicides, causing changes in bee thermoregulation [28]. Azole fungicides inhibit cytochrome P450 detoxification system in bees [7] and thus the bees are more vulnerable to other pesticides.

The octanol-water partition coefficient of tebuconazole (Log P_OW_) has been shown to be 3.7 [29], which means that this compound has lipophilic properties and can concentrate in lipophilic bee matrices. In addition, tebuconazole is quite stable at elevated temperatures and its melting point is 102.4 °C [29], while beeswax melting point is about 63–65 °C [30]. This also confirms that tebuconazole incorporation into molten wax is possible without degradation.

It is vital to understand whether pesticide residues from wax can be taken up by developing honey bees. Medici et al. showed that the presence of insecticides in wax negatively affected honey bee brood survival [31]. There have been only a few studies focused on the migration of pesticides from wax to bees, especially to queen bees. A study conducted by Böhme et al. showed that some pesticide residues (with different Log *p* values) can be found in RJ, though in apparently negligible concentrations (the highest concentration found was 0.016% of the original concentration fed to the nurse bees) [24]. In another experimental study, hives were treated with known amounts of tau-fluvalinate via contaminated plywood inserts, and no residues were detected in RJ [32]. On the contrary, our study revealed that fungicide tebuconazole was translocated from polluted wax to RJ. Nevertheless, tebuconazole concentration found in RJ was 2.4 times lower than in wax. Another study showed that nurse bees fed on contaminated pollen and nectar produced uncontaminated RJ for honey bee queens [33]. Our study revealed that RJ can be contaminated via exposure to residues in wax. Our results are in accordance with Milone and Tarpy who showed that different pesticides mixed into queen cell cups successfully migrated to RJ [34].

Larval feeding pattern performed by nurse bees is different among honey bee castes and sexes (workers, drones, queens). In the case of queen larvae, feeding was performed by nurse bees within relatively short visits and the content of RJ remained almost constant, while worker larvae received longer feeding periods after 48 h [35]. In addition, Dietz and Lambremont showed that honey bee queen larvae consume 13% more food than worker larvae within first the 3 days of larval development [36]. Thus, the higher food consumption by queen bee larvae may simultaneously increase the amount of ingested pesticide residues in the case if RJ is contaminated.

Tebuconazole concentrations found in bee matrices are related to the lipophilicity of this compound, which tends to accumulate in nonpolar media. This tendency was demonstrated in our experiments where the highest concentration was revealed in wax (very non-polar matrix), lower concentration was detected in RJ (up to 6% of fats), and the lowest content in larvae (mostly water-protein sample). Due to the chemical and physical properties of tebuconazole, it preferentially remained in the samples with the highest lipid content. Additionally, tebuconazole has been shown to be persistent in soil (aerobic metabolism T_1/2_ in soils is 796 days) [37]. Even under hydrolytic conditions, it was stable for >28 days. Therefore, the most probable reason for the occurrence of tebuconazole in certain bee products is related to its tendency to stay in a lipophilic environment.

It is noteworthy that there was no conventional farming within 5.4 km radius in our study, which excludes the possibility of bee exposure from agricultural use of tebuconazole. Morales et al. showed that pesticides used on the fields, as well as in apiculture, can move from beeswax to honey bee brood [38]. Interestingly, our results did not indicate tebuconazole residues in the sampled honey bee queen larvae and newly emerged queens. We saw rapid dilution of tebuconazole through different matrices. Compared to the cell cup wax, the whole queen cells contained less than one half the concentration of tebuconazole. Due to the dilution with newly added wax, the initial concentration mixed into queen cell cups had decreased 2.2 times. In addition, the concentration of tebuconazole decreased 2.4 times when comparing RJ to wax. However, if the apiary had been located next to conventional farming lands, the amount of tebuconazole residues in bee matrices could be considerably higher due to the agricultural use of tebuconazole.

Besides chemical decomposition of pesticide molecules over time, the individual bee organisms are also capable of detoxification [39]. In addition, Berenbaum and Johnson have proposed that detoxification of xenobiotic compounds among eusocial honey bees may be complemented by a “social detoxification system”, which includes colony food processing via microbial fermentation, dilution by pollen mixing, and worker discrimination [40]. Without detoxification, the effects of pesticides on honey bee health could be more severe.

Some studies have shown that pesticides may have effects on the longevity, olfactory functions, and water consumption by bees during long-term exposure [41,42]. Nevertheless, in our case, the concentration of tebuconazole after 3 days of exposure was not observed, probably due to the inability of this lipophilic contaminant to migrate from wax to biological tissues of larvae and queen bees.

Tebuconazole concentrations found in bee products in our study probably do not possess any direct lethal effect to developing queens. Still, pesticides even at low concentrations have been shown to cause sub-lethal effects in bees [43]. Fungicide tebuconazole is considered to have low toxicity to bees [44]. Despite the fact that tebuconazole probably does not kill bees directly, it might cause changes in bee homeostasis. As it is known, there are eight different dominant bacterial species in honey bee gut and they tend to exhibit strain diversity according to differences in tolerance of pesticide exposure and metabolic capability [45]. Tebuconazole at high concentrations (5, 50, and 500 mg kg^−1^) has been shown to decrease soil microbial biomass and activity but, interestingly, no clear effect of different concentrations was found [11]. In addition, tebuconazole reduced gut fungal diversity in brown planthopper (*Nilaparvata lugens* Stål). Interestingly, a study conducted by Powell et al. showed that newly emerged honey bees are lacking gut bacteria and their gut is colonized 4–6 days after the emergence via contacts with other workers [46]. Even though tebuconazole does not spread from RJ to bee larvae, a possibility of tebuconazole exposure inside the hive (contaminated pollen, beebread, etc.) cannot be completely excluded. Changes in bee gut microbiota are shown to increase the susceptibility to diseases [47] and bee mortality may be increased due to different sub-lethal effects [45].

## 5. Conclusions

The current pilot study demonstrated the migration of a single pesticide from one bee product to another. Further studies are needed to investigate the potential for the migration of multiple pesticides among bee products, with the goal to identify and quantify the impact on bees associated with possible synergistic and sub-lethal effects.

## Figures and Tables

**Table 1:** Instrumental parameters of the applied method.

Pesticide	Molecular Ion, Da	Daughter Ion, Da	Collision Energy, eV
Tebuconazole	308	70	21
	308	125	34

**Table 2:** Tebuconazole residues detected in bee matrices after spiking queen cell cup wax with tebuconazole at the concentration of 0.412 mg kg^−1^. Additionally, the HQ values of tebuconazole residue in wax are shown. According to the “Bee Tox Wax“ tool data, a HQ value under 250 indicates low wax contamination rate [21].

Bee Matrix.	Control (Tebuco-Nazole Found (mg kg^−1^)	Tebuco-Nazole Found (mg kg^−1^)	% Left from Spiking Concentra-Tion	Tebucona-Zole Contact LD_50_ to Honey Bees 48 h (mg kg^−1^)	Dose (µg) per Bee Found	HQ Value
Queen cells	<0.01	0.19 ± 0.09	46.1	0.2	0.19	1
Royal jelly	<0.01	0.08 ± 0.04	19.4	0.2	0.08	
Larvae	<0.01	<0.01	0	0.2	0	
Queens	<0.01	<0.01	0	0.2	0	

## Data Availability

Data sharing not applicable.
